# Surface Curvature Relation to Protein Adsorption for Carbon-based Nanomaterials

**DOI:** 10.1038/srep10886

**Published:** 2015-06-04

**Authors:** Zonglin Gu, Zaixing Yang, Yu Chong, Cuicui Ge, Jeffrey K. Weber, David R. Bell, Ruhong Zhou

**Affiliations:** 1 Institute of Quantitative Biology and Medicine, SRMP and RAD-X, Collaborative Innovation Center of Radiation Medicine of Jiangsu Higher Education Institutions, Soochow University, Suzhou 215123, China; 2 IBM Thomas J. Watson Research Center, Yorktown Heights, NY 10598, USA; 3 Department of Chemistry, Columbia University, New York, NY 10027, USA

## Abstract

The adsorption of proteins onto carbon-based nanomaterials (CBNs) is dictated by hydrophobic and π-π interactions between aliphatic and aromatic residues and the conjugated CBN surface. Accordingly, protein adsorption is highly sensitive to topological constraints imposed by CBN surface structure; in particular, adsorption capacity is thought to increase as the incident surface curvature decreases. In this work, we couple Molecular Dynamics (MD) simulations with fluorescence spectroscopy experiments to characterize this curvature dependence in detail for the model protein bovine serum albumin (BSA). By studying BSA adsorption onto carbon nanotubes of increasing radius (featuring descending local curvatures) and a flat graphene sheet, we confirm that adsorption capacity is indeed enhanced on flatter surfaces. Naïve fluorescence experiments featuring multi-walled carbon nanotubes (MWCNTs), however, conform to an opposing trend. To reconcile these observations, we conduct additional MD simulations with MWCNTs that match those prepared in experiments; such simulations indicate that increased mass to surface area ratios in multi-walled systems explain the observed discrepancies. In reduction, our work substantiates the inverse relationship between protein adsorption capacity and surface curvature and further demonstrates the need for subtle consideration in experimental and simulation design.

Since the discoveries of fullerene C_60_ in 1985^1^, carbon nanotubes (CNTs) in 1991^2^, and graphene in 2004^3^, carbon-based nanomaterials (CBNs) have attracted considerable attention in various biomedical fields^4^,^5^ (including those related to gene delivery^6^, optical imaging^7^, and nanotherapeutics)^8^,^9^,^10^,^11^,^12^ because of their excellent mechanical, optical, and electrical properties^13^,^14^,^15^. However, growing concerns over pathologies CBNs might effect on human patients have established the study of nanotoxicity as an important element of nanoscience and nanotechnology^16^,^17^,^18^,^19^. Notably, single-wall carbon nanotubes (SWCNTs) have been shown to disrupt the active sites of WW domains and compete with native ligands (proline rich motifs) of SH3 domains. Given the ubiquity and criticality of such proteins in signaling and regulatory pathways^20^,^21^,^22^, interactions between CBNs and WW and SH3 domains are particularly disconcerting. Fullerenes and metallofullerenols have also been shown to inhibit the functions of WW/SH3 domains^17^,^23^ and other proteins like HIV protease^24^. Graphene nanosheets possess the strongest observed capacities for disrupting protein structure among CBNs of various topologies^25^. Furthermore, graphene and graphene derivatives are known to cause extensive damage to cellular membranes, suggesting, in the face of diminished toxicity to eukaryotes, their excellent prospects for use as anti-bacterial agents^26^,^27^,^28^. Various strategies have been proposed to mitigate the cytotoxicity of CBNs to normal cells^29^,^30^ and to target CBN cytotoxicity toward tumor and bacteria cells^31^,^32^. Passivation of CBNs for *in-vivo* applications is commonly achieved through the coating and functionalization of CBN surfaces^33^,^34^,^35^. Many cellular targeting and delivery approaches exploit the adhesion of serum proteins^29^,^30^ to CBNs or make use of CBN aggregation corona that form in the presence of other proteins^36^.

Such interest in designing biocompatible nanoparticles for medical applications has prompted a preponderance of theoretical and experimental studies related to protein-CBN interactions^31^,^37^,^38^. Our previous work suggests that favorable hydrophobic and π-π stacking interactions facilitate the packing of proteins onto CBN surfaces^21^,^22^. Varying topological and chemical features like length^39^, curvature^40^,^41^, surface functionalization^42^ allow CBNs, in principle, to be tuned for specific applications; questions remain, however, concerning just how these properties alter protein adsorption capacity and kinetics. Similarly, it is interesting to consider the role protein structure assumes throughout the adsorption process^43^. CBN surface curvature is generally believed to deprecate adsorption capacity, but, heretofore, systematic studies related to this phenomenon have been sparse.

In this Article, we combine MD simulations with fluorescence spectroscopic measurements to study the adsorption of bovine serum albumin (BSA) onto CBNs with various surface curvatures. Our data derived from MD simulations of SWCNTs support the hypothesized inverse relationship between adsorption capacity and local curvature. However, we find that simple spectroscopic experiments on MWCNTs, when not renormalized for inherent differences in mass-to-surface area ratios, support an inverse trend. To validate this explanation for the observed discrepancy, we conduct additional simulations on several multi-walled CNTs (MWCNTs); we indeed find that increasing mass-to-surface area ratios in larger MWCNTs lead to aberrant reductions in adsorption capacities. Thus, under “corrected” or normalized conditions, we expect both simulation and experiment to confirm that protein adsorptive capacity is enhanced by decrease in surface curvature. The current study illustrates the necessity for designing complementary simulation and experimental protocols, an often non-trivial task in systems existing at the nanoscale.

## Results

### MD simulations reveal the expected adsorption capacity trend with CBN surface curvature

**Figure 1:**
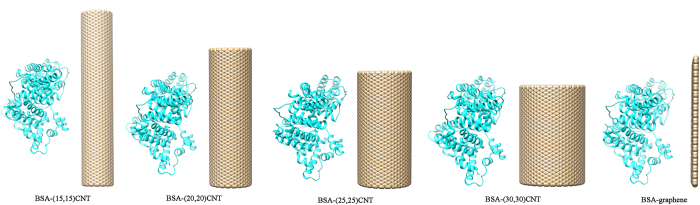
System setup of BSA(cyan) and 5 different CBNs(tan) in the first set of simulations: a (15,15)-SWCNT, a (20,20)-SWCNT, a (25,25)-SWCNT, a (30,30)-SWCNT, and a graphene sheet. Systems are independent but juxtaposed for visualization purposes. At the start of each simulation, the BSA and CBN are separated by 1.0 nm.

In order to investigate the relation between adsorption capacity and surface curvature of CBNs, two sets of five simulation systems were constructed (see Fig. 1 and Fig. S1), each consisting of a BSA protein molecule and a CBN segment featuring a given surface curvature ((15,15)-, (20,20)-, (25,25)-, (30,30)-SWCNTs, and graphene). All systems were started at identical volumes to ensure consistent BSA and CBN concentrations, and the initial minimum distance between the two molecules in each system was set at 1.0 nm. All-atom MD simulations started from each configuration were carried out for a minimum of 200 ns.

**Figure 2:**
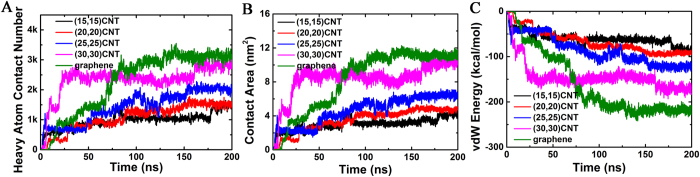
The heavy atom contact number (**A**), contact surface area (**B**) and vdW interaction energy (**C**) between BSA and (15,15)-SWCNT(black), (20,20)-SWCNT(red), (25,25)-SWCNT(blue), (30,30)-SWCNT(magenta) and graphene (green). Collectively, these plots demonstrate that the BSA adsorption capacity increase (measured by more stable adsorption, higher contact numbers, and higher contact surface areas), as the CBN curvature decreases.

**Figure 3:**
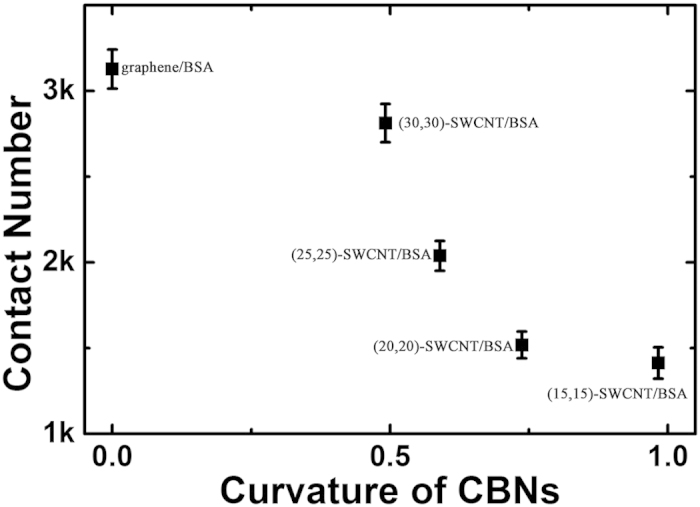
The contact number (heavy atom) versus the surface curvature of CBNs (including all the simulated SWCNTS and graphene). A sigmoidal-like (monotonic but non-linear) decrease of protein adsorption capacity with the increase of the CBN’s curvature can be observed.

Heavy atom contact numbers, contact surface areas, and van der Waals (vdW) interaction energies within each BSA and CBN pair were computed for analysis (Fig. 2). Heavy atom contact numbers (Fig. 2A) indicate that protein adsorption steadily becomes more prominent as the surface curvature of CBNs decreases. After equilibration, average contact numbers corresponding to the graphene and largest-diameter CNT were nearly equal; protein contacts with the narrower CNTs, however, were reduced by a factor of ~2. Contact surface areas and vdW interaction energies exhibited a similar trend (Fig. 2B,C). Results from other simulation trajectories are also consistent (see Fig. S2). To further quantify the relationship between the adsorption capability of CBNs with its curvature, a diagram of heavy atom contact numbers vs curvature was plotted in Fig. 3. Here, the surface curvature was defined as usual:


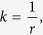


where *k* represents the curvature and *r* represents the radius of the (n,m)-CNT. As shown in Fig. 3, a sigmoidal-like (monotonic but non-linear) dependence of protein adsorption on CBN’s curvature can be observed. Interestingly, the adsorption behavior can be categorized roughly into three curvature regimes (I, 0—0.5 nm^−1^; II, 0.5—0.75 nm^−1^; III, 0.75—1 nm^−1^). In regime I and III, the protein adsorption capability increases slowly with the decrease of the curvature, whereas in regime II, it increases sharply with the decrease of the curvature. These data validate our conjecture that the protein adsorption capacity CBNs should increase with decreasing surface curvature. Notably, the large jumps evident in these property traces correspond to sudden adsorption events seen in our simulations. To better illustrate this phenomenon, two such jumps are exemplified in Fig. S3 (see SI) for the (15,15)-SWCNT/BSA system. From 73 ns to 80 ns, surface contacts involving aliphatic residues Ala581 and Leu582 emerged (as highlighted by the dashed area in Fig. S3A); Fig. S3B shows that a new surface contact involving the aromatic residue Phe501 forms ~180 ns into the simulation. The latter interaction provides an example of π−π stacking between an aromatic side chain and the SWCNT surface; at −6 to −13.5 kcal/mol,^44^ such interactions are thought to be particularly important in adsorption processes.

To further explore the binding mechanism of BSA adsorbed onto the surface of CBNs, the final snapshots for all the five SWCNT systems were examined, with the contact regions highlighted. Meanwhile, all the key residues were identified if their contact probability with CBNs is larger than 0.6, and then further classified into five categories, aromatic, nonpolar, polar, acidic and basic (see Table. S1). As shown in Fig. S4, the exact contact region between BSA and CBNs of various curvatures can be quite different. However, due to the high hydrophobic nature of CBNs, the hydrophobic regions (including nonpolar and aromatic residues) of BSA are more likely to contact with CBNs, despite that the exact contacting residues can be different (Fig. S4 and Table S1).

**Figure 4:**
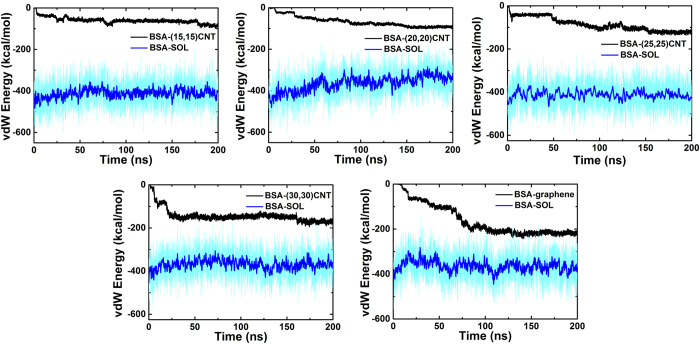
The vdW interaction energy between BSA and CBNs and solvent. The vdW energy between BSA and CBNs and SOL (solvent) are colored in black and cyan (smooth line in blue color). The smoothing was done by moving average with a window size of 50.

To probe trade-offs between adsorption and protein-solvent energetics, we computed the vdW interaction energies corresponding to BSA-CBN and BSA-solvent contacts (Fig. 4). Based on these data, we observe that (i) the vdW energy between BSA and CBNs decreases along the adsorption process (i.e., favorable), and decreases more as the curvature of the CBN decreases and (ii) the vdW energy between BSA and its solvent increases slightly as adsorptive processes occur in simulation. In addition, we also computed the electrostatic interaction energy between BSA and the solvent (Fig. S5), which increases as the simulation time progresses. Clearly, the changes in both the vdW and electrostatic interactions between BSA and the solvent are positive (i.e., unfavorable) for the adsorption of BSA onto the CBNs. In general, it is not the total interaction energy, but the change in the interaction energy that matters. If the energy change during the adsorption process is negative, then this energy component is favorable which helps driving the adsorption process. As we can see from Fig. 4, there is a strong negative change in the vdW interaction between BSA and CNT, thus very favorable for the adsorption process. On the other hand, both the changes in the vdW (Fig. 4) and electrostatic (Fig. S5) interactions between BSA and water are positive and thus unfavorable (due to the removal of water from its interface with CNT once combined). Essentially, the strong and favorable vdW interaction between BSA and CNT drives the adsorption of BSA onto CNT, which partially offsets the net loss in the BSA-water (and CNT-water) interactions. Taken together, the driving force for BSA adsorption is the direct van der Waals (dispersion) interactions between BSA and CBNs, in addition to the hydrophobic interactions (water push both BSA and CBNs together).

### The “validating” fluorescence spectroscopy experiment shows the opposite trend

**Figure 5:**
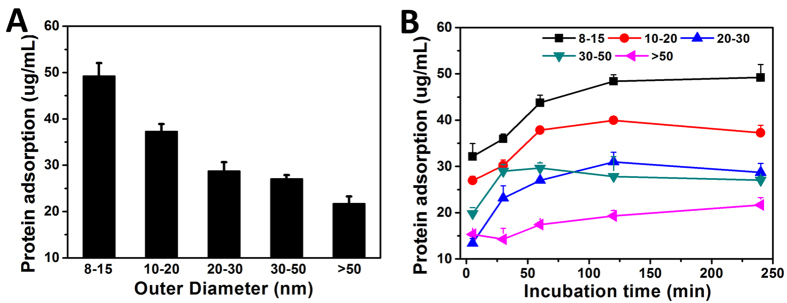
Quantitative analysis of BSA adsorbed by MWCNT nanoparticles with outer diameters of 8–15, 10–20, 20–30, 30–50 and > 50 nm. (**A**) BSA loading capability of each MWCNTs; (**B**) Adsorption kinetic studies of BSA on MWCNTs.

In an attempt to validate our simulation results with experimental data, we prepared five CNT systems with different outer diameters (8–15, 10–20, 20–30, 30–50 and > 50 nm) in fixed concentration suspensions and incubated each system in the presence of excess BSA. To our surprise, the fluorescence data exhibited a trend in complete opposition to our simulation results (Fig. 5A), wherein adsorption capacity seems to decrease with decreasing curvature. Measured kinetic curves (Fig. 5B) also appear to support the opposing adsorption trend.

### Contradiction between experiments and simulations resolved

To reconcile this apparent contradiction, one must make note of a subtle difference between our simulation and experimental protocols. In our simulation systems, the CBNs (SWCNTs, along with graphene) were constrained to have the same mass; in the experimental systems, however, MWCNTs suspensions were prepared at equal concentrations based on the total mass of suspended nanomaterial, due to the inherent difficulty in preparing single-walled CNTs with larger radii in experiment (also the precise size and exact number of walls are still impossible to determine even with today’s state-of-the-art techniques). As the number of layers in a MWCNT increases, its mass increases much more rapidly than its surface area. Accordingly, the adsorption area per unit mass decays as MWCNTs increase in size, explaining the spurious trend observed in our experiment.

**Figure 6:**
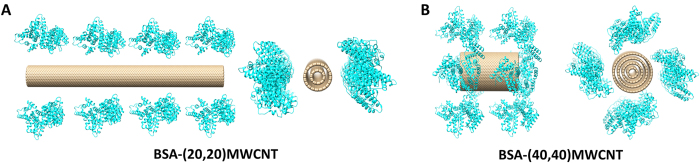
The models of (20,20)-MWCNT/BSAs (**A**) and (40,40)-MWCNT/BSAs (**B**) in original state. BSAs are shown in cyan, the CBNs are shown in tan.

**Figure 7:**
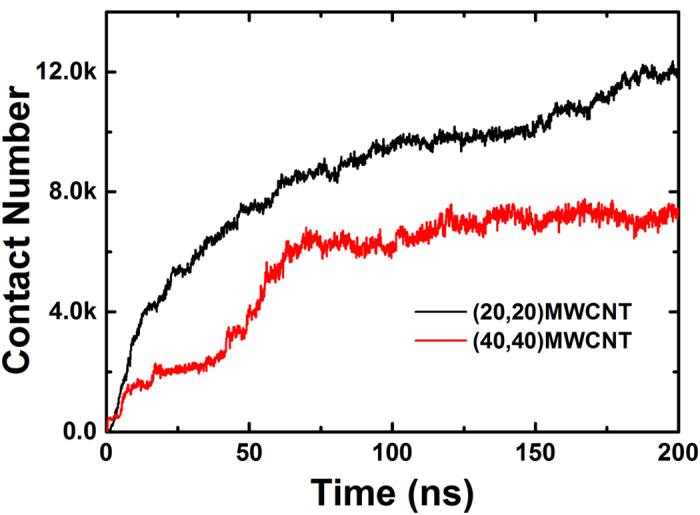
Heavy atom contact number of BSA molecules adsorbing onto two MWCNTs as a function of time. The abscissa represents time and the ordinate represents contact number (in thousands, k  =  1000).

To validate this reasoning, we initiated two respective simulation systems containing BSA and MWCNTs with different radii (Fig. 6). The number of carbon atoms and BSA concentrations are constant in each setup, and the initial CBN-BSA distance was again set to 1 nm. Because of the imposed extensive constraint on the CBNs, the length of (20,20)-MWCNT (27.3 nm) is much longer than that of the (40,40)-MWCNT (8.2 nm), correspondingly the effective surface area of the former (233.2 nm^2^) is much larger than the latter (142.3 nm^2^), and the observed adsorption properties are perturbed accordingly. As illustrated by the heavy atom contact number as a function of the simulation time (Fig. 7), BSA molecules do initially adsorb more quickly onto the (40,40)-MWCNT than onto its more highly curved (20,20)-MWCNT counterpart. However, due to the much larger effective surface adsorption area present in the (20,20)-MWCNT, both such relative adsorbing rates and capacities were quickly reversed. After only about 1.9 ns, the contact number related to the (20,20)-MWCNT already exceeded that of the (40,40)-MWCNT, and after 3.0 ns the adsorption rate of the longer, narrower tube outpaced that of the wider CNT. At simulation’s end, the adsorption capacity of the (20,20)-MWCNT is about twice that of the (40,40)-MWCNT system. Analysis of the contact surface areas and vdW interaction energies exhibited a similar trend as the heavy atom contact number (Fig. S6). Therefore, the results obtained from the simulations on MWCNTs are in excellent agreement with our experimental data.

It should be stressed though that for MWCNTs the effective surface area (the outer surface which could be coated by the proteins) is smaller than SWCNTs with the same mass, which is the quantity used in experiment. The mass to effective-surface area ratio for all the SWCNTs is essentially the same, thus the difference in adsorption capacity is solely due to the surface curvature. While for MWCNTs, the mass to effective-surface area ratio can differ due to the different layers of CNTs contained. Once normalized by this ratio (691.5 and 1147.3 dalton/nm^2^ for (20,20)-MWCNT and (40,40)-MWCNT respectively), the result will show a similar trend with respect to the surface curvature, i.e. the (20,20)-MWCNT will show a lower effective adsorption capacity (contact number ~7,150, averaged over the last 20 ns data, once normalized to the (40,40)-MWCNT value) than the (40,40)-MWCNT (7,180, averaged over the last 50 ns data, which serves as the base here, see Fig. 7), thus consistent with the above single wall results.

Given these supporting simulations, we can confidently argue that the adsorption capacity of proteins on the surface of CBNs increases as the surface curvature decreases. Our study also reinforces, however, the importance of ensuring that underlying simulation models are as consistent as possible with experimental conditions.

## Conclusions

Several theoretical studies^25^,^45^ have shown that protein adsorption onto the surface of CBNs becomes more prominent as the local curvature of the CBNs decreases. Here, we sought to validate this notion using both simulation and experimental methodologies, and, in summary, we succeeded in doing so. But while our initial simulation-based data confirmed the aforementioned hypothesis, seemingly subtle differences in protocol led to drastic deviations between simulation-based and experimental observations that then required additional reconciliation. As the study of nanotoxicity advances in coming years, both simulation and experimental techniques will be of paramount importance; as demonstrated by the present work, however, their harmony must be carefully contrived.

## Methods

### Molecular Dynamics Simulations

All the CBNs coordinates (including (10,10), (15,15), (20,20), (25,25) and (30,30)-SWCNTs, graphene, and (40,40) and (20,20)-MWCNTs) were generated using VMD^46^ (Visual Molecular Dynamics) with all carbon atoms assigned uncharged Lennard-Jones parameters of ε_cc_ = 0.36 kJ/mol and σ_cc_ = 3.4 Å^37,^^47^. (15,15), (20,20), (25,25) and (30,30)-SWCNT had lengths of 12.65 nm, 9.33 nm, 7.49 nm and 6.14 nm with the same carbon atoms number at 3120. The configuration of a SWCNT can be conceptually constructed by “rolling up” a graphene nanosheet into a seamless cylinder. The pattern the graphene is wrapped can be characterized by a pair of indices (*n,m*). The integers *n* and *m* stand for the number of unit vectors along two directions in the honeycomb crystal lattice of graphene. If m = 0, the CNT are called zigzag CNT, and if n = m, they are called armchair CNT. Otherwise, they are called chiral nanotubes. In our study, all the SWCNTs and MWCNTs are the armchair nanotube. The graphene was set to 7.94 nm × 9.95 nm including 3114 carbon atoms (roughly same carbon atoms with CNTs). For all the simulation systems of SWCNTs and graphene, the concentrations of BSA and CBNs were identical. To examine the influence of initial orientation of BSA with respect to the surface of CBNs (SWCNTs and graphene) on the adsorption process and possible binding mechanism, two sets of simulations were carried out for all the five systems, with the protein orientation rotated by 180° (see Fig .1 and Fig. S1, respectively). The (40,40)-MWCNTs had a length of 8.2 nm and consisted of 4 nested layers: (40,40), (30,30), (20,20) and (10,10)-CNTs, specifically. The (20,20)-MWCNT had a length of 27.3 nm and it contained 2 nested layers (composed of (20,20) and (10,10)-CNTs). Both MWCNTs contained the same number of carbon atoms and were subjected to identical BSA concentrations. A crystal structure of BSA (PDB ID: 4F5S^48^) was used to initialize BSA configurations. All the CBNs/BSA complexes were solvated in water boxes, minimized, and equilibrated before production runs in accordance with protocols used in our previous studies^49^,^50^,^51^,^52^,^53^. MD simulations were carried out on a Linux cluster using the software package GROMACS^54^ (version 4.6.4) with CHARMM 27 force field^55^. The temperature and pressure were set at constant to 300 K and 1 bar by using v-rescale thermostat^56^ and Parrinello-Rahman^57^ pressure coupling scheme, respectively. PME (Particle Mesh Ewald)^58^,^59^ was applied to treat long-range electrostatic interactions and the cut-off for handling the van der Waals interactions was set to 10 Å. The TIP3P water model^60^ was used in all simulations.

### Fluorescence spectroscopy

MWCNTs (purity > 90 wt%) with outer diameters of 8-15, 10-20, 20-30, 30-50 and > 50 nm were bought from Chengdu Organic Chemical Company, Chinese Academy of Science. BSA was purchased from Solarbio science &technology (Beijing, China).

MWCNT suspensions (outer diameters of 8-15, 10-20, 20-30, 30-50 and > 50 nm) were prepared by dispersing in pure water, and BSA solutions of the same concentration were prepared in standard phosphate buffer saline (PBS). Then, equal volumes and concentrations (100μg/ml) of MWCNT suspensions and BSA solutions were mixed and shaken at 37 ℃ prior to centrifugation at 14,800 × g for 10 min. After discarding the sediments, the contents of free protein in supernatant were probed by fluorescence spectroscopy. Using the protein standard curve (A = 752.56*C + 20.902, R^2^ = 0.99968, where “A” and “C” represented absorbency and concentration of BSA, respectively), we calculated the percent of protein adsorption on MWCNTs by subtracting the protein content of the supernatant from the total quantity of protein in the system.

## Additional Information

**How to cite this article**: Gu, Z. *et al*. Surface Curvature Relation to Protein Adsorption for Carbon-based Nanomaterials. *Sci. Rep*. **5**, 10886; doi: 10.1038/srep10886 (2015).

## Supplementary Material

Supplementary Information
