# Supplementary material for: Surface Curvature Relation to Protein Adsorption for Carbon-based Nanomaterials
Source: Sci Rep. 2015 Jun 4;5:10886. doi: 10.1038/srep10886 (PMC4455116; doi:10.1038/srep10886)
Supplement: Supplementary Information [file srep10886-s1.pdf]

Surface Curvature Relation to Protein Adsorption for Carbon-based Nanomaterials

Zonglin Gu[1‡], Zaixing Yang[1‡], Yu Chong[1], Cuicui Ge[1], Jeffrey K. Weber[2], David R. Bell[2], and Ruhong Zhou[1,2, 3] *

[1] Institute of Quantitative Biology and Medicine, SRMP and RAD-X, Collaborative Innovation Center of Radiation Medicine of Jiangsu Higher Education Institutions, Soochow University, Suzhou 215123, China

[2] IBM Thomas J. Watson Research Center, Yorktown Heights, NY 10598, USA

[3] Department of Chemistry, Columbia University, New York, NY 10027, USA

‡These authors contribute equally

*Corresponding author, E-mail: ruhongz@us.ibm.com

Supporting information

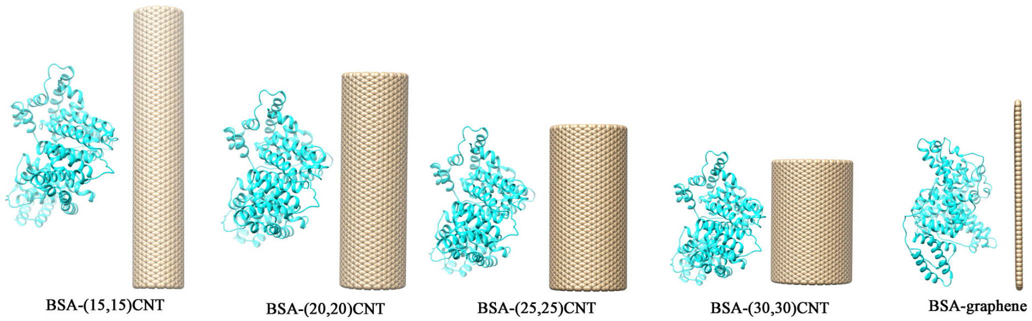

Fig. S1. System setup of BSA(cyan) and 5 different CBNs(tan) in the second set of simulations: a (15,15)-SWCNT, a (20,20)-SWCNT, a (25,25)-SWCNT, a (30,30)-SWCNT, and a graphene sheet. This set of five simulation systems are derived from their corresponding systems in the first set simulations (Figure 1 in the main text) by rotating BSA along the long axis of CBNs by 180°. At the start of each simulation, the BSA and CBN are separated by 1.0 nm.

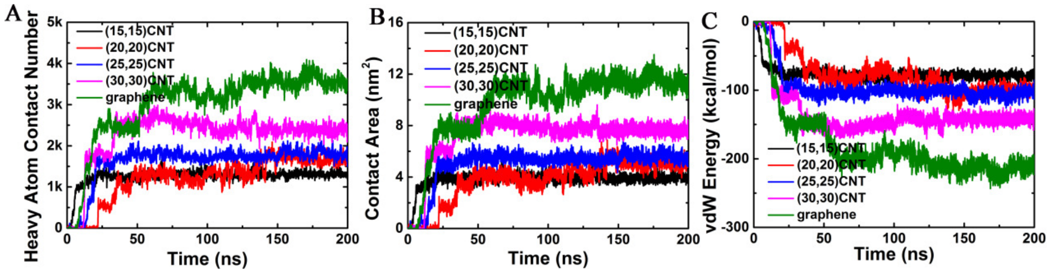

Figure S2. The heavy atom contact number (A), contact surface area (B) and vdW interaction energy (C) between BSA and (15,15)-SWCNT (black), (20,20)-SWCNT (red), (25, 25)-SWCNT (blue), (30,30)-SWCNT (magenta) and graphene (green) for the second set of simulation on the SWCNTs. Collectively, these plots further confirm that the BSA adsorption capacity increases as the CBN curvature decreases.

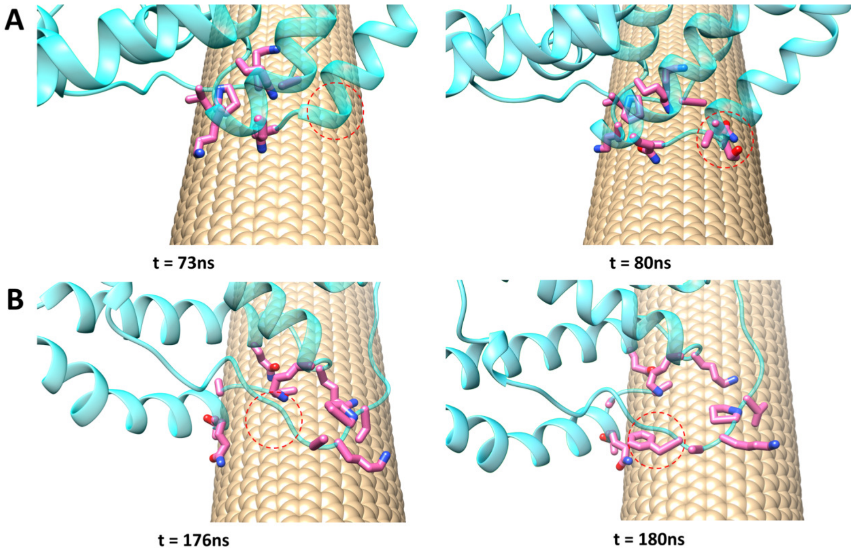

Fig. S3 Local snapshots of BSA binding to (15,15)-SWCNT in two time frames, where there are large jumps in the contact number and contact surface area. The red rounded dashed areas indicate the contact sites of BSA forming these emerged new contacts (Ala581, Leu582 in A; Phe501 in B).

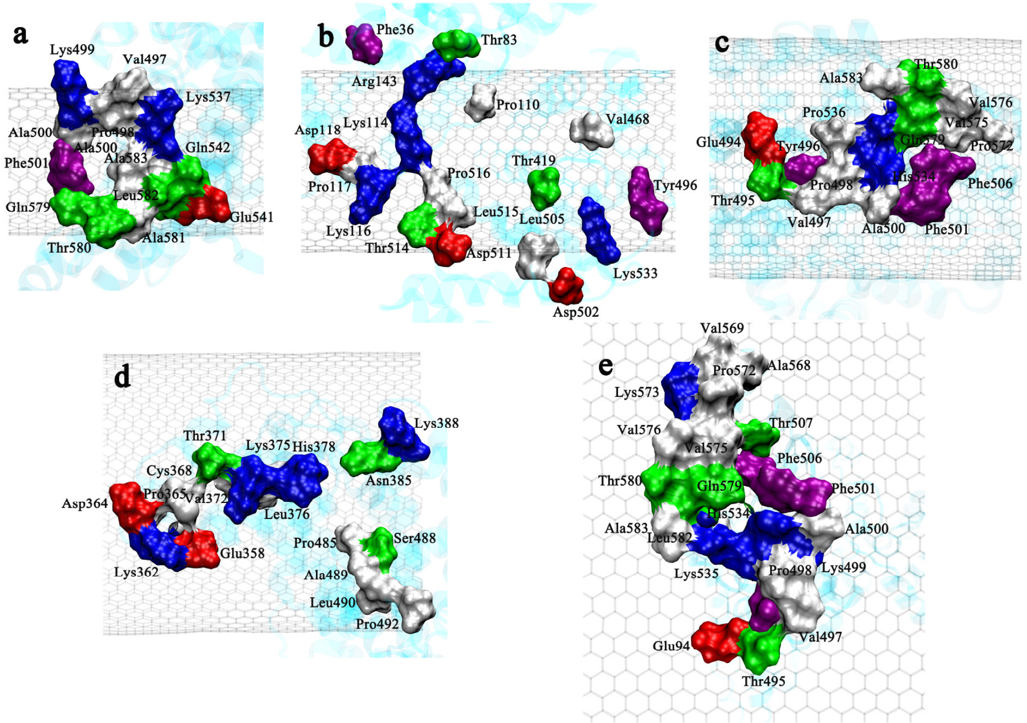

Fig. S4. The final snapshots for all the five SWCNT systems in the first set simulations. The CBNs are shown in transparent tan bonds. The adsorption region of BSA onto the surface of CBNs are colored in terms of the residue types, with nonpolar, aromatic, acidic (negatively

charged), basic (positively charged) and polar residues colored in white, purple, red, blue and green, respectively.

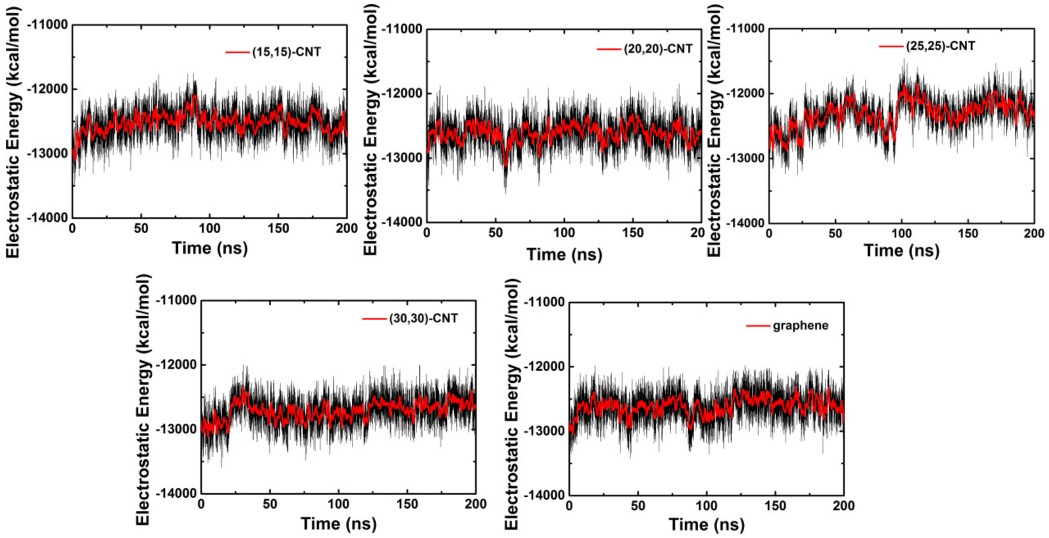

Fig. S5. The electrostatic interaction energy between BSA and the solvent. The electrostatic interaction potential energy between BSA and SOL (solvent) are colored in black (smooth line in red color). The smoothing was done by moving average with a window size of 50.

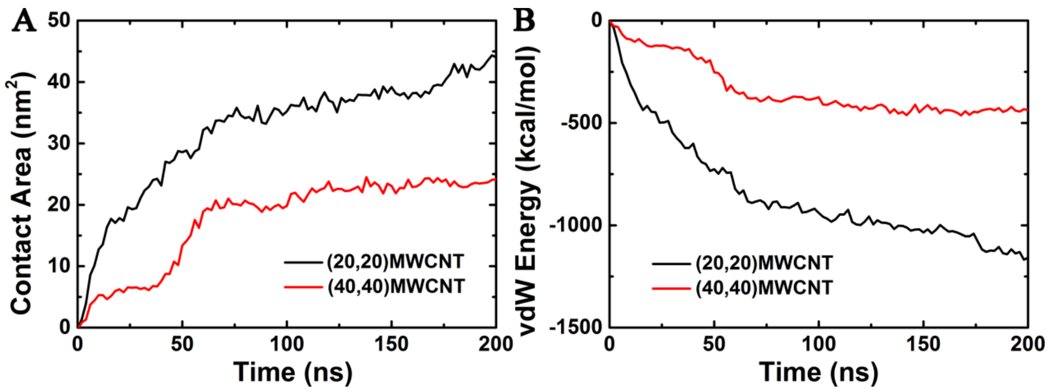

Fig. S6. The contact surface area (A) and vdW interaction energy (B) between BSA and (20,20)-MWCNT(black), and (40,40)-MWCNT(red). The contact surface areas and vdW interaction energies exhibited a similar trend as the heavy atom contact number, shown in Figure 7 of the main text.

Table. S1. Distributions of the key residues that are in tight contact with CBNs in terms of the properties of the residue side chains (residue types).

	Aromatic	Basic	Acidic	Nonpolar	Polar
(15,15)CNT-BSA	1	2	1	7	3
(20,20)CNT-BSA	2	3	1	6	2
(25,25)CNT-BSA	3	3	1	8	3
(30,30)CNT-BSA	0	6	2	9	5
Graphene-BSA	3	4	1	10	4
